# The somatic mutation profile of estrogen receptor-positive HER2-negative metastatic breast cancer in Brazilian patients

**DOI:** 10.3389/fonc.2024.1372947

**Published:** 2024-06-17

**Authors:** Tomás Reinert, Fernanda Orpinelli do Rego, Matheus Costa e Silva, Amanda Muniz Rodrigues, Fernanda Christtanini Koyama, Aline Coelho Gonçalves, Maiane Maria Pauletto, Leandro Jonata de Carvalho Oliveira, Cristiano Augusto Andrade de Resende, Luciana Castro Garcia Landeiro, Carlos Henrique Barrios, Max Senna Mano, Rodrigo Dienstmann

**Affiliations:** ^1^ Oncoclínicas & Co, São Paulo, Brazil; ^2^ Grupo Brasileiro de Estudos em Câncer de Mama (GBECAM), Porto Alegre, Brazil; ^3^ Oncoclínicas Precision Medicine (OCPM), São Paulo, Brazil; ^4^ University of Vic – Central University of Catalonia, Vic, Spain

**Keywords:** breast neoplasm, estrogen receptor, genomic landscape, PI3K/Akt pathway, targeted therapies

## Abstract

**Background:**

Breast cancer is the leading cause of cancer death among women worldwide. Studies about the genomic landscape of metastatic breast cancer (MBC) have predominantly originated from developed nations. There are still limited data on the molecular epidemiology of MBC in low- and middle-income countries. This study aims to evaluate the prevalence of mutations in the PI3K-AKT pathway and other actionable drivers in estrogen receptor (ER)+/HER2- MBC among Brazilian patients treated at a large institution representative of the nation’s demographic diversity.

**Methods:**

We conducted a retrospective observational study using laboratory data (OC Precision Medicine). Our study included tumor samples from patients with ER+/HER2- MBC who underwent routine tumor testing from 2020 to 2023 and originated from several Brazilian centers within the Oncoclinicas network. Two distinct next-generation sequencing (NGS) assays were used: GS Focus (23 genes, covering *PIK3CA*, *AKT1*, *ESR1*, *ERBB2*, *BRCA1*, *BRCA2*, *PALB2*, *TP53*, but not *PTEN*) or GS 180 (180 genes, including PTEN, tumor mutation burden [TMB] and microsatellite instability [MSI]).

**Results:**

Evaluation of tumor samples from 328 patients was undertaken, mostly (75.6%) with GS Focus. Of these, 69% were primary tumors, while 31% were metastatic lesions. The prevalence of mutations in the PI3K-AKT pathway was 39.3% (95% confidence interval, 33% to 43%), distributed as 37.5% in *PIK3CA* and 1.8% in *AKT1*. Stratification by age revealed a higher incidence of mutations in this pathway among patients over 50 (44.5% vs 29.1%, p=0.01). Among the *PIK3CA* mutations, 78% were canonical (included in the alpelisib companion diagnostic non-NGS test), while the remaining 22% were characterized as non-canonical mutations (identifiable only by NGS test). *ESR1* mutations were detected in 6.1%, exhibiting a higher frequency in metastatic samples (15.1% vs 1.3%, p=0.003). Additionally, mutations in *BRCA1, BRCA2*, or *PALB2* were identified in 3.9% of cases, while mutations in *ERBB2* were found in 2.1%. No *PTEN* mutations were detected, nor were TMB high or MSI cases.

**Conclusion:**

We describe the genomic landscape of Brazilian patients with ER+/HER2- MBC, in which the somatic mutation profile is comparable to what is described in the literature globally. These data are important for developing precision medicine strategies in this scenario, as well as for health systems management and research initiatives.

## Introduction

Breast cancer (BC) is the most commonly diagnosed malignancy and the leading cause of cancer death among women worldwide, with 60% of BC-related mortality occurring in low- to middle-income countries (LMIC) (1).

Translational research efforts have brought extensive knowledge about the molecular epidemiology of BC in different disease scenarios. Nevertheless, studies about the genomic landscape of metastatic breast cancer (MBC) have predominantly originated from developed nations (2). Studying the molecular epidemiology of BC globally is crucial for several reasons, including heterogeneity of the genetic background, environmental exposures, lifestyle, and healthcare access of different populations that contribute to variations in the incidence and molecular characteristics of the disease. Notably, attributes of Brazilian patients with BC like African and Latin ancestries, elevated BC incidence in young women, and a higher proportion of advanced stage at diagnosis may be related to distinct clinicopathological and molecular epidemiology profiles (3).

Estrogen receptor-positive (ER+) and HER2-negative (HER2-) tumors are the most common subtype of BC and are responsible for most of the deaths from the disease. Breast cancers are known to undergo genomic evolution during the course of the disease, with the acquisition of genotypic and phenotypic alterations associated with resistance to therapeutic strategies leading to disease progression (4). Significant advances have been made in understanding the molecular complexity that governs the interplay between the ER pathway and pivotal growth factors, metabolic, and cell division signaling. This knowledge opens avenues for optimizing therapeutic outcomes through the manipulation of endocrine signaling and intervention in diverse mechanisms of endocrine therapy (ET) resistance (5).

Notable advancements include emerging therapeutic agents such as the oral selective ER degrader (SERD) elacestrant, PI3K inhibitors alpelisb and inavolisib, and the AKT inhibitor capivasertib. These drugs have exhibited clinical efficacy by extending progression-free survival (PFS) in phase III clinical trials, specifically benefiting patients with identifiable biomarkers such as *ESR1* mutations and molecular alterations in the PI3K-AKT pathway, respectively (6, 7). Simultaneously, there is growing interest in tumor-agnostic treatment strategies, underscoring the importance of global molecular epidemiology studies (8). Examples include mutations in *BRAF* V600E or microsatellite instability (MSI). The identification of genomic biomarkers in patients with ER+/HER2- MBC becomes increasingly important in tailoring therapeutic approaches, ensuring precision in treatment modalities, and contributing to the evolution of personalized medicine (9).

This study aims to evaluate the genomic landscape of ER+/HER2- MBC among Brazilian patients treated at a large institution representative of the nation’s demographic diversity. Our objective is to characterize the molecular epidemiology of ER+/HER2- MBC in this population, highlighting existing and emerging genomic biomarkers of interest for developing precision oncology, with particular interest in the prevalence of mutations within the PI3K-AKT pathway.

## Materials and methods

We conducted a retrospective observational study using a laboratory cohort. Our study includes tumor samples from patients with ER+/HER2- MBC who underwent routine tumor testing between June 2020 and June 2023. All tumors were tested in a single reference laboratory (OC Precision Medicine) and originated from several Brazilian centers within the Oncoclinicas network. The molecular profiling was carried out as part of routine care and funded by internal resources (institutional funding) or external partners (pharmaceutical patient support programs). To be eligible for testing, patients had either *de novo* metastatic disease or relapsed disease. Samples from both primary tissue and metastatic lesions were eligible for testing.

### Sample collection

The Institutional Ethics Committee approved the study, and patients signed an informed consent form for molecular testing that allows the analysis of aggregated de-identified data for research purposes. The consent form does not cover access to clinical data (only demographic and histopathology information is available in the test request).

Assuming a prevalence of 40% of molecular alterations in the PI3K-AKT pathway, a sample size of 350 cases provides 5% absolute precision in the estimate with a 95% confidence interval. Fisher’s exact test was used for exploratory subgroup comparisons of the prevalence of gene alterations according to patient age (younger or older/equal to 50 years) and biopsy site (primary or metastatic lesion), with P value < 0.05 being statistically significant.

Appropriate formalin-fixed paraffin-embedded (FFPE) tissue was defined as >20% tumor cells and <10% necrosis. DNA was extracted using ReliaPrep FFPE (Promega). For GS 180, the minimum DNA amount was 200 ng, and RNA was 250 ng. For GS Focus a minimum of 40 ng was required.

### Sequencing

Two distinct next-generation sequencing (NGS) assays were employed using the QIAseq Targeted DNA Custom Panel (QIAGEN). Detailed information on gene and exon coverage can be found in Supplementary Data.

GS 180 has full exon coverage of *PIK3CA*, *AKT1*, *ESR1*, *ERBB2*, *BRCA1*, *BRCA2*, *PALB2*, *TP53*, as well as *PTEN*. TMB was estimated with GS180 panel, with 15 mutations/megabase as a validated cut-off for high TMB (using FoundationOne CDx as gold-standard assay). Sequencing was performed in the Illumina NovaSeq platform.

The targeted GS Focus panel covers single nucleotide variants (SNV) and insertion/deletion (Indel) in 23 cancer genes, including *PIK3CA*, *AKT1*, *ESR1*, *ERBB2*, *BRCA1*, *BRCA2*, *PALB2*, *TP53*, but not *PTEN*. Sequencing was performed in the Illumina MiSeq platform.

### Sequencing data analysis and variant detection

The sequencing data (paired end reads 2x150) were analyzed using CLC Genomics Workbench (Qiagen) for GS Focus using the pipeline developed for QIAseq Targeted DNA Panels (Qiagen), and the variants were confirmed by experts using QIAGEN Clinical Insight (QCI). This test has been internally validated to detect SNV and Indel variants at 5% allele frequency or higher in target regions with sufficient read coverage (>100x). The broad somatic panels GS180 (180 cancer genes) use Anchored Multiplex PCR (AMP) methodology based on the multiplex polymerase chain reaction (PCR) developed by ARCHER for DNA sequencing, including SNV, Indel, copy number variation (CNV), microsatellite instability (MSI) and tumor mutational burden (TMB) analysis, coupled with RNA sequencing with FusionPlex Solid Tumor kit for gene fusions and rearrangements. MSI Sensor v2 algorithm was used to detect MSI high (10). The sequencing data (paired end reads 2x150) are aligned to the hg19 human genome reference using the Novoalign tool (11). A variant calling approach using MuTect2, LoFreq, GATK, and a hotspot caller developed in the laboratory was applied by the experts to detect SNV, CNV, and Indel variants (12, 13). This test has been validated to detect SNV and Indel variants at 5% allele frequency or higher in target regions with coverage of enough reading (> 100 Mean coverage collapsed).

We also investigated the additional value of NGS for *PIK3CA* mutation detection when compared with the coverage of the polymerase chain reaction (PCR) companion diagnostic test for alpelisib (*therascreen* PIK3CA RGQ PCR Kit). Both PCR and NGS detected Category 1 mutations, while Category 2 mutations are non-canonical oncogenic alterations covered by NGS alone and companion diagnostic test used for capivasertib in Capitello-291 (14).

## Results

Of 350 ER+/HER2- MBC patients eligible for testing during the study period, 328 (94%) tumor samples had informative NGS results. All patients were women. As described in **Table 1**, 69% of the samples originated from primary tumors, while 31% were from metastatic sites. The median age of patients was 58 years, with 110 (33.5%) being younger and 218 (66.4%) older than 50. Most patients were from the Southeast region (62.8%), but patients from the Northeast (18%), South (11.5%) and Midwest (8%) regions from Brazil were also represented.

**Table 1:** Overall description of patient and tumor sample characteristics (n=328).

Variable		N (%)
Age	<50 years	110 (33.5%)
	>=50 years	218 (66.5%)
Sex	Female	328 (100%)
Biopsy site	Primary tumor	225 (68.6%)
	Metastatic lesions	93 (28.4%)
	Unknown	10 (3%)
NGS panel	GS Focus	248 (75.6%)
	GS 180	80 (24.4%)
Geographic region	Southeast	206 (62.8%)
	Northeast	59 (18%)
	South	38 (11.5%)
	Midwest	25 (8%)

**Figure 1:**
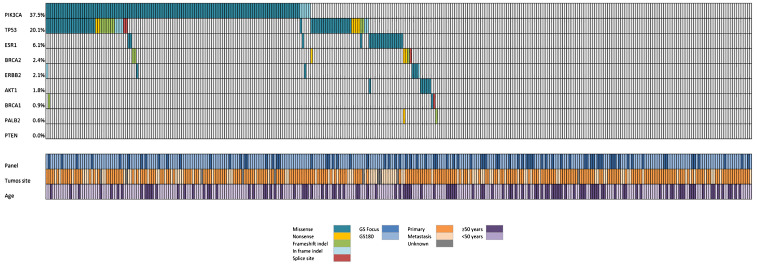
Oncoprint showing the distribution of genomic alterations according to NGS panel, tumor site, and patients’ age.

**Figure 2:**
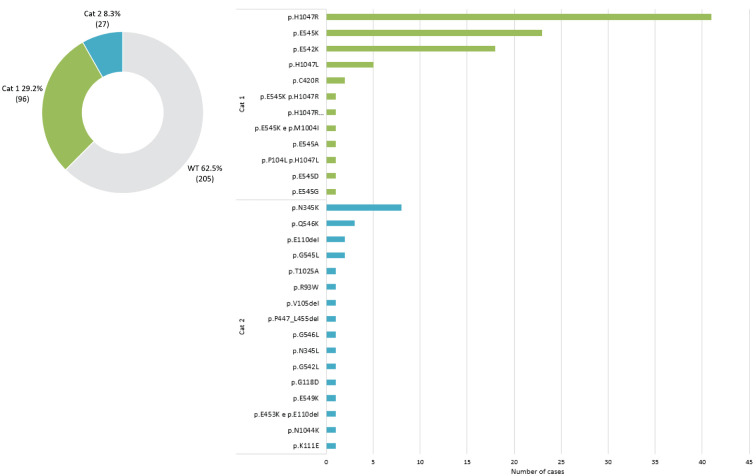
PIK3CA mutational status distribution according to OncoKB categories (14).

**Figure 1** illustrates the molecular epidemiology profile of the cohort. The prevalence of mutations in the PI3K-AKT pathway was 39.3% (95% CI 34%-44%), distributed as 37.5% in *PIK3CA* and 1.8% in *AKT1*. We found no mutations in *PTEN* in 80 samples sequenced with GS180 panels. Stratification by age revealed a higher prevalence of mutations in the PI3K-AKT pathway among patients over 50 years (44.5% vs 29.1%, P=0.01). There was no difference in *PIK3CA* mutation rate comparing primary and metastatic tumor samples (37.3% vs 36.6%, P=0.99). Among the *PIK3CA* mutations, 78% were canonical (included in the *therascreen* companion diagnostic PCR kit), with the remaining 22% were non-canonical mutations (not identifiable by non-NGS testing), as illustrated in **Figure 2**.

*ESR1* mutations were detected in 6.1% of the samples, exhibiting a higher frequency in metastatic lesions than in primary sites (15.1% vs 1.3%, respectively, P=0.003). Additionally, somatic alterations in *BRCA1*, *BRCA2*, and *PALB2* were identified in 3.9% of cases, while mutations in *ERBB2* were found in 2.1%. No mutations were identified in *BRAF* V600E. Also, we detected no gene fusions in *NTRK1–3* or *RET*, and no cases had MSI high or TMB high covered by GS180.

## Discussion

Therapeutic algorithms are evolving in MBC with the approval of innovative therapies and the greater incorporation of precision medicine concepts, for which access to NGS panels is essential. As one example, therapies targeting endocrine resistance mutations in the PI3K-AKT pathway (15). The Cancer Genome Atlas (TCGA) initially described the profile of somatic mutations in MBC in primary tumors (16). Subsequently, a series of research studies described the molecular profile of breast cancer patients in a range of MBC patient scenarios, such as *de novo* MBC and ER+ MBC resistant to endocrine therapy (17–19).

For various reasons, such as well-established research infrastructures (academic intuitions and high-throughput technologies) and funding, most molecular epidemiology studies have been conducted in high-income countries (HIC). There is growing recognition of the need for more research in LMIC. The Brazilian population has unique ethnic characteristics and is considered an “ethnic melting pot,” reflecting an admixture of European, Native American, and Sub-Saharan African people, in addition to immigrants from Asian countries. Recent publications have described the germline molecular epidemiology of BC in Brazil, demonstrating unique genetic features such as the Brazilian *TP53* R337H variant, detected in 1.6% of BC patients and 0.1% of reference controls, strongly associated with risk of BC (OR = 17.4, p<0.0001) (20).

However, there is still a lack of data evaluating the somatic mutational profile in MBC. As an example, recent data presenting the somatic mutation profile of BC in Uganda revealed that some characteristics were similar to European patients (such as the prevalence of *PIK3CA* mutation of 39%), while other features were characteristic in patients with African Ancestries and nearly half of the women had either a mutation in *BRCA1* (24%) and *BRCA2* (24%) (21). Pan et al. reported that compared to breast tumors in Caucasian women, there is an increased prevalence of HER2-enriched molecular subtypes and a higher prevalence of *TP53* somatic mutations in ER+ Asian breast tumors (22). Women from Latin America were underrepresented in the TCGA analysis, where only 31 out of approximately 1,100 total BCs are from Latinas. Neuhausen et al. evaluated the somatic tumor profile in 142 Latinas with invasive BC and showed that the somatic mutation rates were comparable to European patients, but trends were observed in genes more commonly mutated in Latinas, such as *PIK3CA* (23, 24).

The PI3K-AKT pathway is recognized as one of the most critical mechanisms of endocrine resistance. Mutations in *PIK3CA* tend to be truncal and are consistently present in approximately 30–50% of patients with MBC (25). Although they are not relevant for clinical decisions in initial and first-line disease, the mutational status of this pathway is a biomarker for the use of different second-line therapies such as the PI3K inhibitors alpelisib (26) (based on SOLAR-1 data in case of canonical mutations in *PIK3CA*) and the AKT inhibitor capivasertib (based on Capitello-291 data in case of oncogenic molecular alterations in *PIK3CA*, *AKT1* or *PTEN*). Furthermore, recent studies have shown positive results with the PI3K inhibitor inavolisib in combination with a CDK4/6 inhibitor plus ET as first-line therapy for MBC. This may modify the therapeutic algorithm and promote the earlier need for somatic sequencing (27). Notably, the somatic NGS panel can offer other information with clinical relevance, such as the presence of mutations in *BRCA1*, *BRCA2*, *PALB2*, *ERBB2*, and *ESR1*, in addition to molecular changes that can be treated with tumor-agnostic therapies.

Our study describes the molecular landscape of Brazilian patients with ER+/HER2- MBC in a context representative of standard clinical practice. Tissue samples from primary and metastatic tumors were evaluated, and the mutation profile was comparable to the data described in the literature. The prevalence of mutations in the PI3K-AKT pathway was 39%, and this proportion is important not only from a medical point of view but also impacts health systems management and clinical research initiatives.

One important finding of our study is that 22% of the mutations in *PI3KCA* are non-canonical oncogenic mutations, which would not be identifiable by the alpelisib PCR-based companion test, emphasizing the importance of NGS panels to expand the identification of PI3K-AKT pathway alterations. Similarly, Martínez-Saez et al. evaluated data from over 6,000 patients with breast cancer, explored across 10 publicly available studies, and reported that around 20% of *PIK3CA* mutations would not have been detected by the therascreen PCR-based companion test (28). The low frequency of mutations in *ESR1* is explained by our cohort’s low representation of metastatic tissue. Despite a series of methodological limitations and the fact that not all samples were evaluable for these markers, we did not find actionable fusions, TMB high, or MSI in any case, which suggests the limited applicability of the concept of tumor-agnostic therapies in standard clinical practice for ER+/HER2- MBC (8).

Our study presents a series of limitations, such as its retrospective nature, the lack of clinical correlations, treatments used and outcomes, the lack of matching with germline sequencing, and the heterogeneity of clinical scenarios since most of the analyses were carried out on tissue from the primary tumors in patients that developed metachronous metastases. Also, we did not have longitudinal matched samples for intra-patient comparisons. Finally, our sample size allowed high precision for mutations in the 40% range (such as PI3K-AKT pathway) but not for rare mutations or signatures, such as TMB high.

Nevertheless, our study presents several strengths, such as the representativeness of the Brazilian BC patient population seen in standard clinical practice, the fact that all samples were analyzed in one central laboratory using validated NGS assays, the use of broad panels covering the full range of biomarkers used in breast oncology.

Understanding the molecular complexity of diseases, particularly in diverse populations such as Brazilian MBC patients, is essential for devising interventions and treatments with broader relevance. Initiatives are underway to bridge existing disparities, foster inclusivity in research, and guarantee that the insights gained from molecular epidemiology studies extend to a broader spectrum of cancer patients. These endeavors encompass enhancing translational research capabilities, cultivating international collaborations, and expanding access to clinical research in LMIC (29).

## Conclusion

Our manuscript reveals the genomic landscape of Brazilian patients with ER+/HER2- MBC. The profile of somatic mutations in the PI3K-AKT pathway was similar to that described in the literature. The study identifies considerations for diagnostic testing, particularly regarding non-canonical *PIK3CA* mutations that mandate broad exon coverage with NGS assays. While our study lacks clinical correlates, it contributes with valuable insights to the understanding of molecular alterations in Brazilian MBC patients, highlighting the clinical relevance of the PI3K-AKT pathway.

## Data availability statement

The datasets presented in this study can be found in online repositories. The names of the repository/repositories and accession number(s) can be found in the article/**Supplementary Material**.

## Ethics statement

The studies involving humans were approved by Instituto oncoclinicas - The study was approved by the Ethics Committee protocol CAAE: 75984723.0.0000.0227. The studies were conducted in accordance with the local legislation and institutional requirements. The participants provided their written informed consent to participate in this study.

## Author contributions

TR: Conceptualization, Data curation, Methodology, Supervision, Validation, Visualization, Writing – original draft, Writing – review & editing. FO: Data curation, Formal analysis, Methodology, Resources, Supervision, Validation, Visualization, Writing – original draft, Writing – review & editing. MS: Data curation, Formal analysis, Methodology, Software, Validation, Writing – review & editing. AR: Data curation, Formal analysis, Methodology, Supervision, Writing – review & editing. FK: Conceptualization, Data curation, Methodology, Project administration, Resources, Supervision, Validation, Writing – original draft, Writing – review & editing. AG: Writing – original draft, Writing – review & editing. MP: Writing – original draft, Writing – review & editing. LO: Writing – original draft, Writing – review & editing. CR: Conceptualization, Data curation, Formal analysis, Funding acquisition, Investigation, Methodology, Project administration, Resources, Software, Supervision, Validation, Visualization, Writing – original draft, Writing – review & editing. LL: Writing – original draft, Writing – review & editing. CB: Writing – original draft, Writing – review & editing. MM: Writing – original draft, Writing – review & editing. RD: Writing – review & editing, Conceptualization, Data curation, Formal analysis, Investigation, Methodology, Project administration, Resources, Supervision, Validation, Visualization, Writing – original draft.
