# Supplementary material for: The somatic mutation profile of estrogen receptor-positive HER2-negative metastatic breast cancer in Brazilian patients
Source: Front Oncol. 2024 Jun 17;14:1372947. doi: 10.3389/fonc.2024.1372947 (PMC11215150; doi:10.3389/fonc.2024.1372947)
Supplement: Supplementary file 1 [file DataSheet_1.pdf]

SUPPLEMENTARY MATERIAL

REFERENCE LIST OF GENES INCLUDED IN THE NGS PANELS USED IN THE STUDY

GS Focus
Gene (exons)

a) SNV/Indel: ALK (21-28), AKT1 (3, 6), ATM (full exon), BRAF (7, 11, 12, 15, 16), BRCA1 (full exon), BRCA2 (full exon), EGFR (4, 6, 7, 12, 15-23, 25), ERBB2 (full exon), ESR1 (8), FGFR3 (3, 6- 9, 14-16, 18), HRAS (2-5), IDH1 (3, 4), IDH2 (4), KIT (2, 8- 11, 13-15, 17, 18, 20, 21), KRAS (2-5), MET (14), NRAS (2-4), PALB2 (full exon), PDGFRA (3- 5, 7, 10, 12-15, 18, 19, 23), PIK3CA (full exon), POLE (9-14, 21, 25), RET (10, 11, 13-16),TP53(fullexon)

GS180
Gene (exons)

a) SNV/Indel: ABL1 (4-7), AKT1 (3, 6), AKT2 (2-14), AKT3 (1-13), ALK (1, 21-28), APC (2, 10, 12, 16), AR (1-8), ARID1A (1-20), ATM (1), ATR (1-47), ATRX (1-35), AURKA (2, 5-8), B2M (1-3), BAP1 (1-17), BARD1 (1-11), BRAF (11, 15), BRCA1 (1-23), BRCA2 (1-27), BRIP1 (2-20), CCND1 (1-5), CCND2 (1-5), CCNE1 (2-12), CD274 (1-7), CDH1 (1-16), CDK4 (2-7), CDKN2A (1-3), CDKN2B (1), CHD1 (1-35), CHEK1 (2-13), CHEK2 (2-15), CIC (1-20), CSF1R (7, 22), CTNNB1 (3), DAXX (1-8), DDR2 (3, 12-18), DDX3X (1-17), EGFR (1-28), EP300 (1-31), ERBB2 (8, 10, 19-21, 24), ERBB3 (1-3, 7-8, 28), ERBB4 (3-4, 6-9, 15, 23), ERCC2 (1-23), ESR1 (8), EZH2 (16), FBXW7 (1-11), FGF19 (3), FGFR1 (3, 7-8, 13, 15, 17), FGFR2 (7, 9, 12, 14), FGFR3 (3, 6-9, 14-16, 18), FLT3 (11, 14, 16, 20), FOXA1 (1-2), FOXL2 (1), FUBP1 (1-19, 21), GNA11 (5), GNAQ (4-5), GNAS (1, 6-9), H3-3A (), H3-3B (2-4), HNF1A (3-4), HRAS (2-3), IDH1 (3-4), IDH2 (4), IGF1R (1-5, 8-21, 67), JAK1 (2-25), JAK2 (3-25), JAK3 (2-18, 20-24), KDM6A (1-29), KDR (6-7, 11, 19, 21, 26-27, 30), KEAP1 (2-6), KIT (2, 8-11, 13-15, 17-18), KLF4 (1-5), KMT2C (1-59), KRAS (2-5), LZTR1 (1-21), MAP2K1 (2-3, 6-7), MAP3K1 (1-20), MDM2 (2-4, 6, 8, 10), MDM4 (4, 11), MED12 (1-45), MEN1 (2-10), MET (2, 11, 14, 16, 19-21), MLH1 (1-19), MPL (10), MRE11 (2- 19), MSH2 (1-16), MSH6 (1-10), MYC (1-3), MYCL (1-3), MYCN (3), NBN (1-16), NF1 (1-57), NF2 (1-16), NKX2-1 (1-2), NOTCH1 (25-27, 34), NOTCH2 (34), NPM1 (11), NRAS (2-5), PALB2 (1-13), PBRM1 (2-30), PDGFRA (3, 11-12, 14-15, 18, 23), PIK3CA (2, 5, 7-8, 10, 14, 19, 21), PIK3R1 (1-10), PMS2 (1-15), POLE (1-49), PPP2R1A (1-15), PRKCA (1-17), PRKD1 (1-18), PTCH1 (1-23), PTEN (1-9), PTPN11 (3, 13), RAD50 (1-25), RAD51B (2-11), RAD51C (1-9), RAD51D (1-10), RAD54L (1-18), RB1 (1-27), RET (10-11, 13-16), RHOA (2-3), RNF43 (2-10), ROS1 (36-40), SDHB (1-8), SETD2 (1-21), SMAD2 (7), SMAD4 (2-12), SMARCA4 (2-29, 31-35), SMARCB1 (2, 4-5, 7, 9), SMO (3, 5-7, 9, 11), SRC (14), STAG2 (2-32, 35), STK11 (1-9), SUFU (1-12), SUZ12 (1-16), TERT (1, 6), TGFBR2 (3), TP53 (1), TP63 (1-14), TRAF7 (2-21), TSC1 (3-23), TSC2 (2-42), TSHR (9), VHL (1-3), XRCC2 (1-3), XRCC3 (4-10)
b) CNV: ABL1, AKT1, AKT2, AKT3, ALK, APC, AR, ARID1A, ATM, ATR, ATRX, AURKA, B2M, BAP1, BARD1, BRAF, BRCA1, BRCA2, BRIP1, CAMTA1, CCNB1, CCND1, CCND2, CCND3, CCNE1, CD274, CDH1, CDK4, CDK6, CDKN2A, CHD1, CHEK1, CHEK2, CIC, CSF1R, CTNNB1, DAXX, DDR2, DDX3X, E2F2, EGFR, EP300, ERBB2, ERBB3, ERBB4, ERCC2, ESR1, EZH2, FBXW7, FGF19, FGFR1, FGFR2, FGFR3, FLT3, FOXA1, FOXL2, FUBP1, GLI2, GNA11, GNAQ, GNAS, H3-3B, HNF1A, HRAS, IDH1, IDH2, IGF1R, JAK1, JAK2, JAK3, KDM6A, KDR, KEAP1, KIT, KLF4, KMT2C, KRAS, LZTR1, MAP2K1, MAP3K1, MDM2, MDM4, MED12, MEN1, MET, MITF, MLH1, MPL, MRE11, MSH2, MSH6, MYC, MYCL, MYCN, NBN, NF1, NF2, NKX2-1, NOTCH1, NOTCH2, NRAS, PALB2, PBRM1, PDGFRA, PIK3CA, PIK3R1, PLAUR, PMS2, POLE, PPP2R1A, PRKCA, PRKD1, PTCH1, PTEN, PTPN11, RAD50, RAD51B, RAD51C, RAD51D, RAD54L, RB1, RET, RHOA, RNF43, ROS1, SDHB, SETD2, SMAD4, SMARCA4, SMARCB1, SMO, STAG2, STK11, SUFU, SUZ12, TERT, TP53, TP63, TRAF7, TSC1, TSC2, VHL, XRCC2, XRCC3

c) Fusions (RNA): AKT3 (1-3), ALK (19-22), AR (1-8), ARHGAP26 (2, 10-12), AXL (19), BRAF (7-12, 15), BRD3 (9-12), BRD4 (10-11), CSF1 (5-9), CSF1R (7, 11-13, 22), EGFR (7-9, 16, 20, 24-25), ERG (2-11), ESR1 (3-6), ETV1 (3-13), ETV4 (2, 4-10), ETV5 (2-3, 7-9), ETV6 (1-7), EWSR1 (4-14), FGFR1 (2, 8-10, 17), FGFR2 (2, 8-10, 17), FGFR3 (8-10, 17), FGR (2), INSR (12-22), JAZF1 (2-4), MAML2 (2-3), MAST1 (7-9, 18-21), MAST2 (2-3, 5-6), MET (2, 13-16), MSMB (2-4), MUSK (7, 9-10, 12-15), MYB (7-9, 11-16), NOTCH1 (2, 4, 26-31), NOTCH2 (5-7, 26-28), NRG1 (1-3, 6), NTRK1 (8, 10-13), NTRK2 (11-17), NTRK3 (13-16), NUMBL (3), NUTM1 (3), PDGFRA (7, 10-15), PDGFRB (8-14), PIK3CA (2), PKN1 (10-13), PPARG (1-3), PRKCA (4-6), PRKCB (3), RAF1 (4-7, 9-12), RELA (3-4), RET (8-13), ROS1 (31-37), RSPO2 (1-2), RSPO3 (2), TERT (2), TFE3 (2-8), TFEB (1- 2), THADA (24-31, 36-37), TMPRSS2 (1-6).

d) Signatures: microsatellite instability (MSI based on MSI Sensor v2) and tumor mutation burden (TMB based on internal pipeline, high if > 15 mutations/megabase).
